# Maternal and Fetal Outcomes After Interferon Exposure During Pregnancy: A Systematic Review With Meta-Analysis

**DOI:** 10.3389/frph.2021.702929

**Published:** 2021-08-12

**Authors:** Mengmeng Zhang, Shan Fu, Danfeng Ren, Yuchao Wu, Naijuan Yao, Tianzhi Ni, YaLi Feng, Yaolong Chen, Tianyan Chen, Yingren Zhao, Jinfeng Liu

**Affiliations:** ^1^Department of Infectious Diseases, The First Affiliated Hospital of Xi'an Jiao Tong University, Xi'an, China; ^2^Department of Infectious Diseases, Xi'an, China; ^3^Evidence-Based Medicine Center, Basic Medical Sciences, Lanzhou University, Lanzhou, China; ^4^WHO Collaborating Center for Guideline Implementation and Knowledge Translation, Lanzhou, China

**Keywords:** interferon, pregnancy, outcomes, systematic review, meta-analysis

## Abstract

Interferon (IFN) treatment is widely applied in viral hepatitis and multiple myeloproliferative diseases. However, there is considerable controversy on how to deal with unintended pregnancy during IFN treatment, even selective termination is suggested by hepatologists. To settle this clinical dilemma, we conducted a systematic review to retrieve all published articles involving IFN exposure during pregnancy up until March 31, 2021. Only 8 case reports that were relevant with outcomes of pregnant women with viral hepatitis exposed to IFN-α were retrieved, and 17 studies reporting pregnancy outcomes after exposure to type I IFNs involving 3,543 pregnancies were eligible for meta-analysis. No birth defect was reported in the case reports of pregnant women with viral hepatitis. The meta-analysis showed that risks of pregnancy outcomes and birth defects were not increased after exposure to IFN-α. Further comprehensive meta-analysis concerning the IFN-α and IFN-β exposure demonstrated that the risks of live birth (OR 0.89, 95% CI: 0.62–1.27), spontaneous abortion (OR 1.09, 95% CI: 0.73–1.63), stillbirth (OR 1.38, 95% CI: 0.51–3.72), preterm delivery (OR 1.24, 95% CI: 0.85–1.81), and maternal complications (OR 0.72, 95% CI: 0.38–1.38) were not increased in patients exposed to IFNs. The pooled estimates of live birth, spontaneous abortion, stillbirth, preterm delivery, and maternal complications were 85.2, 9.4, 0, 7.5, and 6.5%, respectively. Importantly, the risk of birth defects was not increased (OR 0.68, 95% CI: 0.39–1.20) after IFN exposure, with a pooled rate of 0.51%. Therefore, IFN exposure does not increase the prevalence of spontaneous abortion, stillbirth, preterm delivery, and birth defects. Clinical decision should be made after weighing up all the evidence.

## Introduction

As a pivotal cause of liver cirrhosis and hepatocarcinoma, chronic hepatitis B virus (HBV) infection remains a major health problem with global impact and accounts for approximately one million deaths annually, which surpasses the deaths caused by human immunodeficiency virus, tuberculosis, and malaria infection (1). Antiviral therapy against HBV has displayed significant effectiveness in improving survival and quality of life by preventing disease progression, and even reversing liver fibrosis and cirrhosis (2). In May 2016, the World Health Organization proposed the goal for elimination of viral hepatitis as a public health threat by 2030. In this instance, aggressive antiviral treatment is suggested. According to some guidelines, an increasing number of female patients initiate antiviral treatment in their childbearing age (2, 3).

Since be cloned and purified, interferon (IFN) is the first approved biotherapeutic agent. With accumulating studies, IFNs present not only antiviral activity with a spectrum of clinical effectiveness, but are also the prototypic biological response modifiers for cancers. Currently, genetically engineered IFNs have been applied primarily in the treatment of patients with HBV and hepatitis C virus infection, multiple sclerosis (MS), malignant melanoma, neuroendocrine tumors, and certain lymphoproliferative and hematological diseases (4). It is now well established that type I IFN can induce innate immune function and inhibit HBV replication (5, 6). Since type I IFN can induce long-term immunological control of HBV replication within a finite duration treatment, sets of guidelines have recommended type I IFN as preferred initial therapy (2, 7, 8). Moreover, with accumulated data about antiviral effectiveness of IFN, an expert consensus has recommended combined therapy of IFN and nucleos(t)ide analogs to peruse functional cure for chronic HBV patients (9). Therefore, IFN has been extensively applied to treat chronic HBV patients for clinical cure, especially in the young population.

With the latest estimate, there are 65 million women of reproductive age with HBV infection (1). Globally, 44% of pregnancies are unintended. Although effective contraception is required during IFN therapy, unintended pregnancy is still inevitable (10). IFN was contraindicated during pregnancy considering the potential influence on fetuses. Whereas, among the majority of guidelines on the management of HBV infection, there are few recommendations about how to deal with unintended pregnancy during IFN treatment (2, 7, 11, 12) or even termination of pregnancy is suggested in Chinese guidelines (13). Nevertheless, it is the concern of obstetricians that the significant high prevalence of secondary infertility would lead to depression and discrimination (14). Given these potential challenges, counseling about the possible risks to fetuses after IFN exposure is an important part of disease management for chronic HBV patients. At present, there is no consensus on the management of unintended pregnancy in female patients with HBV infection during IFN therapy.

This highlights the need for up-to-date information on IFN safety during pregnancy. We found low-quality evidence about the safety of IFN during pregnancy; randomized controlled study of IFN administration during pregnancy is unlikely to be conducted given the ethical concerns of such a trial. Compared with any single study, meta-analysis has more power for statistical performance, and increased precision for confidence intervals, because the conclusions often reflect a broad spectrum of objectives and study characteristics and are more generalizable. Consequently, meta-analysis may be an appropriate alternative to help guide decision making in this special situation. Furthermore, since type I IFN has also been approved for MS (15), essential thrombocythemia (ET) (16), and other myeloproliferative diseases, systematic evaluation of all information about IFN exposure during pregnancy would provide a comprehensive safety profile for hepatologists.

Therefore, we conducted this meta-analysis of all available studies to evaluate the effect of IFN on pregnancy outcomes and provided references for clinical decisions.

## Materials and Methods

The systematic review and meta-analysis were registered in advance in PROSPERO (CRD42020183239) and were reported in line with the guidelines of Preferred Reporting Items for Systematic Reviews and Meta-Analyses (PRISMA) (17) and Meta-analysis of Observational Studies in Epidemiology (MOOSE).

### Literature Search Strategy

We searched MEDLINE (*via* PubMed), the Cochrane Library, Embase, the China National Knowledge Infrastructure Database, Wanfang Databases, Database of Chinese Technical Journal, and Chinese Biology Medicine database from inception to March 31, 2021. We designed an extensive search strategy, included Medical Subject Headings words and free keywords with the following terms: (“maternal” OR “gravidity” OR “mother/mothers” OR “gravidities” OR “pregnant women” OR “pregnancy”) AND (“interferon” OR “interferon-*” OR “pegylated interferon” OR “peginterferon”). In addition, to ensure all relevant articles were captured, two independent reviewers (MMZ and SF) conducted a manual search of reference lists and bibliographies of the included articles, as well as references from international meetings, original articles, clinical guidelines, narrative reviews, and previous systematic reviews or meta-analysis. There was no date or geographic restrictions, and translators were used when applicable.

### Study Selection

Any original published human studies fulfilling the following criteria were included in the meta-analysis:

**Population:** Female patients received IFN treatment at any dose within 1 month before conception or at any time of pregnancy.

**Intervention/exposure:** IFN treatment/exposure at any dose within 1 month prior to conception or at any time of pregnancy.

**Comparison:** Pregnant women with the same diseases without IFN exposure.

**Outcomes:** Pregnancy outcomes, including but not limited to live birth, spontaneous abortion, stillbirth, preterm delivery, maternal complications, and birth defects.

We excluded studies in which: (1) patients received other potentially teratogenic medications prior to or during pregnancy; (2) duplicate publications and publications including data from the same patients; and (3) published as conference, review, or abstract.

### Data Management and Extraction

Literature search was performed by two independent investigators (MMZ and SF), as well as the data were strictly evaluated according to the inclusion and exclusion criteria. Discrepancies were resolved by consulting with the third investigator (JFL). The following information was collected using a customized data extraction form followed by cross-checking: first authors, published year, study region, study design, sample size, basic disease, obstetric history, period of pregnancy when exposed to IFN, duration of IFN exposure, the type and dose of IFN exposure, infant information, and maternal and fetal outcomes.

### Definition of Outcomes

We set the possible pregnancy outcomes: live birth, spontaneous abortion, stillbirth, and preterm delivery. According to the European Medicines Agency (EMA) and Centers for Disease Control and Prevention (CDC) guidelines, a live birth was defined as any delivery resulting in viable neonates after 24 weeks of gestation; spontaneous abortion was defined as spontaneously ended pregnancy before 22 weeks of gestation (according to EMA guidelines) or 20 weeks of gestation (according to CDC guidelines), including abortion, miscarriage, missed abortion, incomplete abortion, and early fetal death; stillbirth was defined as a fetal death occurring after 22 weeks of gestation (according to EMA guidelines) or 20 weeks of gestation (according to CDC guidelines), further defined as fetal death or loss before or during delivery; and preterm delivery was defined as any delivery of viable neonates too early, before 37 weeks of gestation.

The major outcome concerned with fetuses/neonates was birth defect, which was defined as a morphological, functional, and/or biomedical developmental disturbance before birth, at birth, or any time after birth. In addition, maternal complications were also recorded in terms of Reproductive Health from CDC (18).

### Quality Assessment

Two researchers used Newcastle–Ottawa Scale (NOS) to assess the bias risk of observational studies. In this scale, studies were scored on the information of selection of study groups (0–4 stars), comparability (0–2 stars), and outcome ascertainment (0–3 stars).

### Statistical Analysis

We calculated the odds ratios(ORs)and 95% confidence intervals (CIs) to compare the risk of different outcomes between IFN-exposed and unexposed groups. We also performed a proportion meta-analysis to calculate the pooled rate of each outcome in patients exposed to IFN, which was estimated using the number of events divided by the total number of pregnancies. The estimates were put in context with the general population using the published data. The heterogeneity of studies was assessed by a Chi-square test and *I*^2^ statistics; *I*^2^ > 50% was considered as significantly heterogeneous. According to the differences of results and *I*^2^ of the two methods, the random effect model or the fixed effect model was applied to combine the results of the studies. Publication bias was calculated by visual funnel plot and assessed by Begg's test and Egger's test. *P* < 0.05 was considered statistically significant. All analyses were performed by STATA (Version 13).

## Results

### Characteristics of Identified Studies

**Figure 1:**
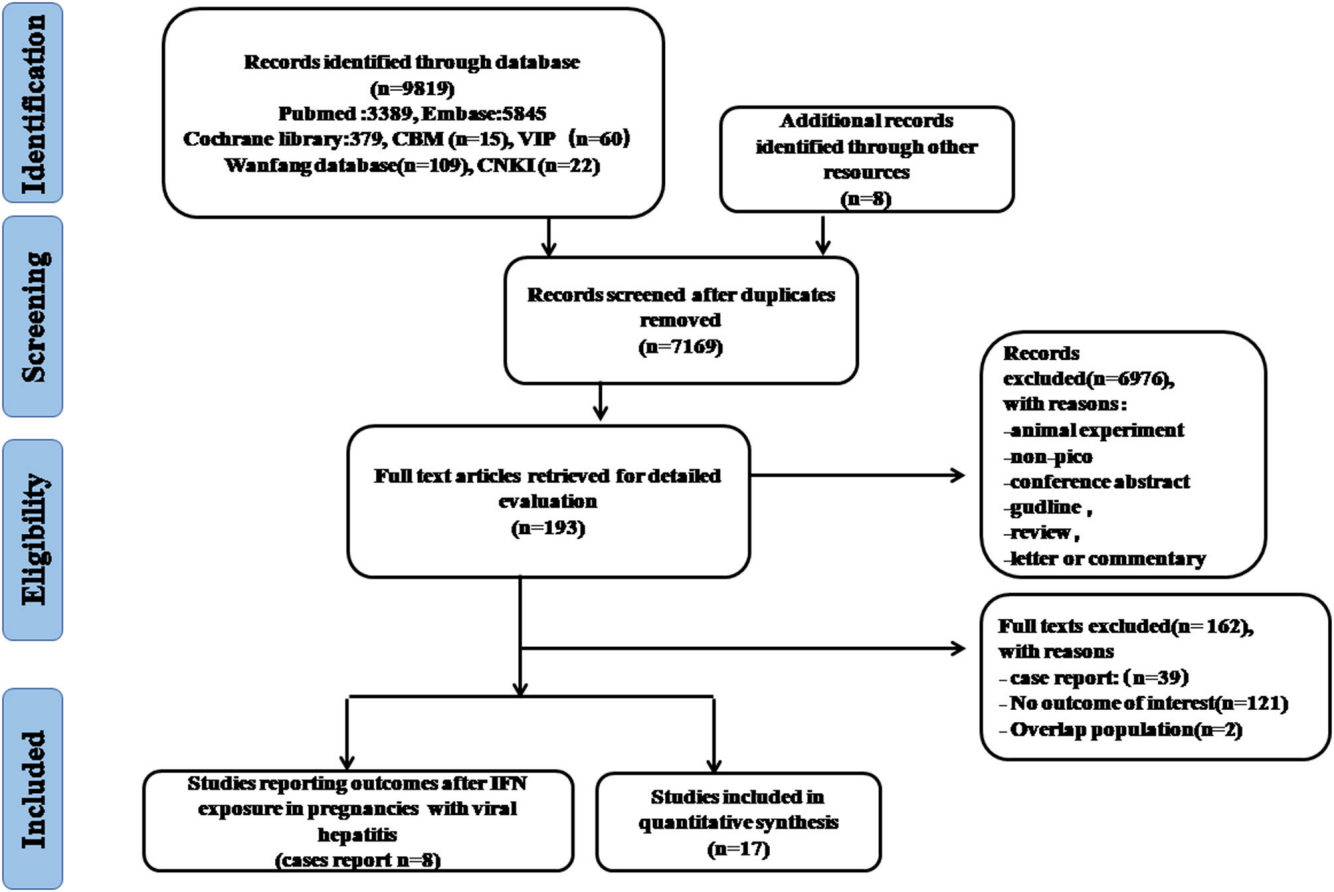
Flowchart of study selection.

As showed in the flowchart of study selection (Figure 1), our search strategy yielded 9,819 citations preliminarily. After removing duplications and other studies through reading the title and abstract, 193 articles were then reviewed by full-text assessment, and 17 studies covering more than 10 countries (United States, Italy, Germany, Canada, United Kingdom, France, Brazil, Japan, Sweden, Australia, European Economic Area and Global database) were included in the final meta-analysis. There were only eight case reports on pregnancy outcomes after IFN-α exposure in women with viral hepatitis.

Five studies reporting pregnancy outcomes after IFN-α exposure were eligible for meta-analysis, in which the enrolled patients suffered from multiple myeloproliferative diseases (Table 1). Considering IFN-β, another type I IFN was also approved for chronic HBV treatment (35), there were 12 studies eligible to evaluate pregnancy outcomes after IFN-β exposure. The main characteristics of the included studies for meta-analysis are detailed in Table 1. The quality of the eight cohort studies was assessed with NOS scores, whereas four studies were at a low risk of bias (Supplementary Table). In total, 3,543 pregnancies were included for final analysis, among which 2,462 pregnancies were exposed to IFN before conception or during pregnancy and 1,081 were not exposed to IFN.

**Table 1:** Characteristics of the 17 included studies for meta-analysis.

**References**	**Study country**	**Study type**	**Study period**	**Basic disease**	**Duration of therapy**	**Type of IFN**	**Age, y, mean (range)/mean±SD**	**Pregnancy outcomes #**	**Pregnancies in exposed group**	**Pregnancies in unexposed group**	**NOS**
Weber, Schaefer (19)	Germany	Prospective	1996–2007	MS	First trimester	IFN-β	30	①②③④⑥	69	64	9
Amato, et al. (20)	Italian	Prospective	2001–2008	MS	4.6 ± 5.8 weeks	IFN-β	32.6 (±4.8)	①②③④⑤⑥	88	318	8
Boskovic, et al. (21)	Canada	Prospective	1997–2004	MS	9 weeks (ranging from 2 to 38 weeks)	IFN-β	35.6 ± 5.3	①②④⑤⑥	23	21	8
Thiel, et al. (22)	German	Prospective	2008–2013	MS	32 days	IFN-β	31.54	①②③④⑥	251	194	6
Patti, et al. (23)	Italy	Retrospective	1997–2006	MS	9.1 ± 1.9 weeks	IFN-β	34.8 ± 4.2	①②③④⑤⑥	14	24	8
Lu, et al. (24)	Canada	Prospective	1998–2009	MS	Within 1 month prior to conception and/or during pregnancy	IFN-β	24.4 ± 5.7	①②③⑥	15	317	5
Sandberg, et al. (25)	Sweden	Retrospective	1994–2003	MS	Within 2 weeks prior to conception and/or during pregnancy	IFN-β	30.3 (25-38)	①②③④⑤⑥	41	6	6
Melillo, et al. (26)	Italian	Retrospective	1998–2007	ET	Full term	IFN-α	33	①②③④⑥	20	102	6
Cincotta, et al. (27)	Australia	Retrospective	1988–1998	ET	Not described in detail	IFN-α	30.7	①②④⑥	4	26	NA
Beauverd, et al. (28)	United Kingdom	Retrospective	2013–2015	ET	Full term	PEG-IFN	32	①②③④	10		
Sandberg, et al. (29)	Global	Prospective	1998–2009	MS	Within 2 weeks prior to conception and/or during pregnancy	IFN-β	NA	①②③⑥	425		
Romero, et al. (30)	Global	Prospective	1993–2013	MS	Not described in detail	IFN-β	31.2	①②③④⑥	423		
Hellwig, et al. (51)	European Economic Area	Retrospective	2009–2017	MS	First trimester	IFN-β	NA	①②③④⑥	948		
Shimizu, et al. (31)	Japan	Retrospective	2006–2010	MS	Not described in detail	IFN-β	30	①②③④⑥	21		
Lapoirie, et al. (32)	France	Retrospective	2004–2014	MPN	Not described in detail	IFN-α	NA	①②③④⑤⑥	6		
Moura, at al. (33)	Brazil	Retrospective	2000–2016	CML	Not described in detail	IFN-α	29	①②③④⑤⑥	9		
Coyle, et al. (34)	United States	Prospective	2006–2012	MS	Not described in detail	IFN-β	30.0 ± 5.29	①②③④⑥	96		

# *①live birth, ②spontaneous abortion, ③stillbirth, ④preterm delivery, ⑤maternal complications, ⑥birth defects (all)/congenital anomalies*.

*MS, multiple sclerosis; ET, essential thrombocythemia; MPN, myeloproliferative neoplasms; CML, chronic myeloid leukemia*.

### Systematic Analysis of Outcomes in Pregnant Women With Viral Hepatitis Exposed to IFN-α

There were no high-quality controlled clinical trials and cohort studies retrieved, which were concerned with unintended pregnancy during IFN-α treatment of viral hepatitis. With primary search, there were only eight case reports about the outcomes of pregnant women with viral hepatitis exposed to IFN-α. As presented in Table 2, all of those pregnant women navigated safely throughout the pregnancy and no birth defects were reported associated with IFN exposure.

**Table 2:** Characteristics of cases reported with pregnancy outcomes after IFN-α exposure in patients with viral hepatitis.

**Reference**	**Year**	**Country**	**Age**	**Type of IFN**	**Disease**	**Exposed period**	**Outcome**	**Exposed details**
Hiratsuka, et al. (36)	2000	Japan	44	IFN-α	HCV	13th week to 33rd week of gestation	healthy male infant	5 MU, 2–4 times a week, a total dose of 315MU
Suda, et al. (37)	1999	Japan	21	IFN-α	HCV	Third trimester of pregnancy (half of year)	healthy female infant	2.5MU, 3 times per week for half a year
Atasoy, et al. (38)	2017	Turkey	39,28	PEG IFNα-2b	HBV	first trimester	healthy infant	10th week to16th week of gestation
Özaslan, et al. (39)	2002	Turkey	26	IFN-α	HCV	from 16th week of gestation	healthy infant	3 MU, 3 times per week for 2.5 months
Makin, et al. (40)	2013	Japan	25	IFN-α	HBV	during the second trimester	healthy female infant	3MU, 2 weeks during the second trimester
Ruggiero, et al. (41)	1996	Italy	42	IFN-α	HCV	the first 5 months of gestation	healthy female infant	3 MU, 3 times per week
Trotter, et al. (42)	2001	United States	27	IFN-α	HCV	first trimester	healthy female infant	3 MU, 3 times per week
Daniel, et al. (43)	2001	Canada	29	IFN-α-2b	HCV	became pregnant 3.5 months after discontinuing treatment	healthy female infant	3 MU, 3 times per week

### Meta-Analysis of Pregnancy Outcomes After IFN-α Exposure

Considering the application of IFN-α in multiple myeloproliferative diseases for more than 30 years, the effect was further assessed in pregnant women with multiple myeloproliferative diseases exposed to IFN-α. As shown in Figure 1, five studies were eligible to evaluate the safety of IFN-α exposure and pooled estimate of each pregnancy outcome.

**Figure 2:**
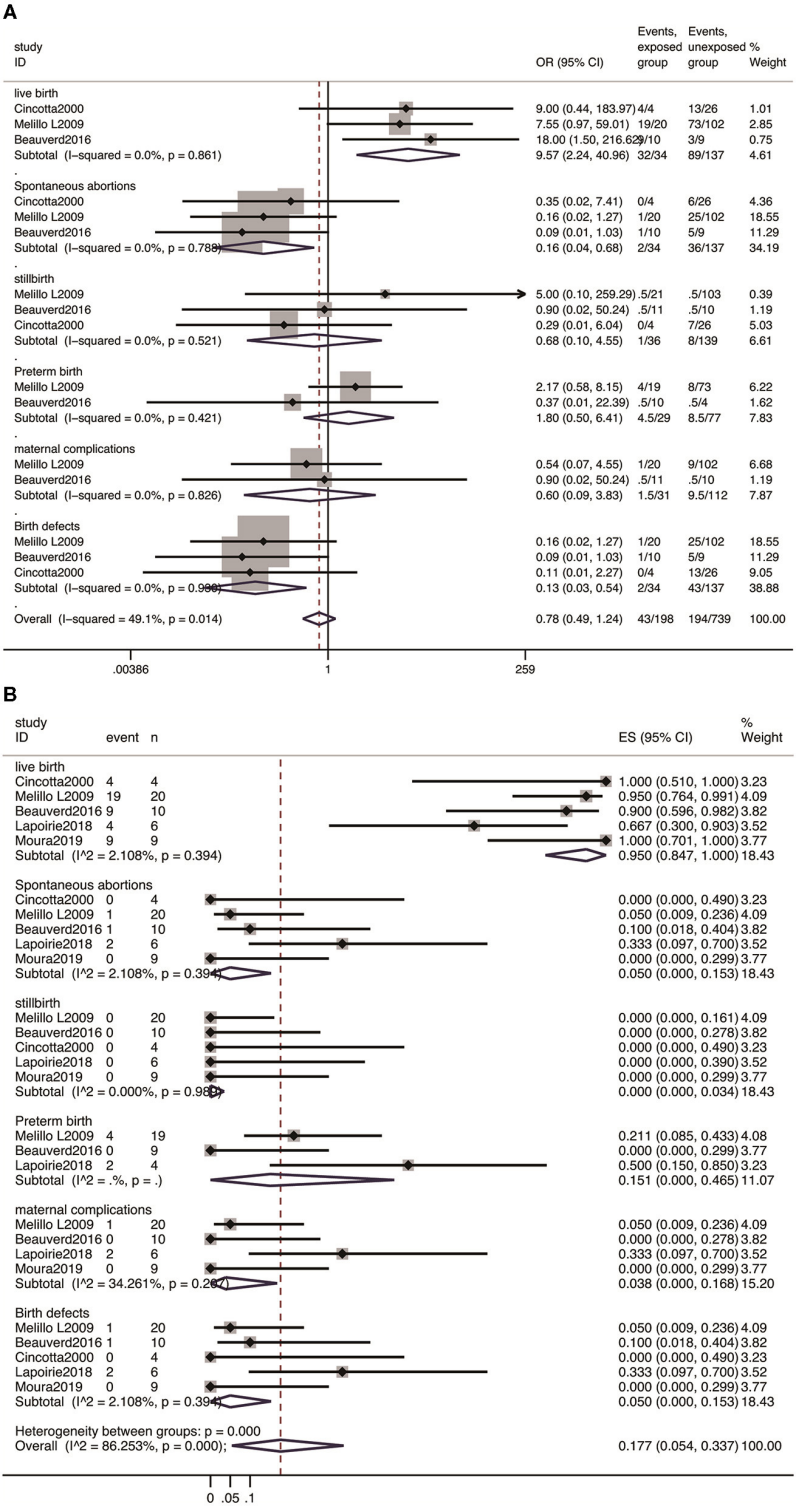
Meta-analysis of pregnancy outcomes with IFN-α exposure. **(A)** The OR of pregnancy outcomes in IFN-α-exposed group compared with un-exposed group; **(B)** the pooled rate of each pregnancy outcome in IFN-α-exposed patients.

In line with our expectations, the majority of the outcomes were normal live births. As shown in Figure 2, the prevalence of live birth was 95.0% (95% CI: 0.847–1.000). Compared with patients unexposed to IFN-α, it showed that the rate of live birth was increased with IFN-α treatment (OR 9.57, 95% CI: 2.24–40.96; *P* = 0.861). The analysis showed no increased risk of stillbirth (OR 0.68, 95% CI: 0.10–4.55, *P* = 0.69) and preterm birth (OR 1.80, 95% CI: 0.50–6.41, *P* = 0.368). The risk of spontaneous abortion was reduced with IFN-α treatment (OR 0.16, 95%CI: 0.04-0.68, *P* = 0.013), which was attributed to the effectiveness of IFN-α treatment. Furthermore, the studies yielded a pooled prevalence of 5.0% (95% CI: 0.00-0.153) in spontaneous abortion, 15.1% (95% CI: 0.00–0.465) in preterm abortion, and 0% (95% CI: 0.00–0.034) in stillbirth.

Among 49 pregnancies exposed to IFN-α in these five cohorts, no birth defects were reported, the pooled rate was 0% (95% CI: 0.000–0.068), and the OR for the estimate of birth defects after IFN exposure was 2.0 (95% CI: 0.11–34.94, *P* = 0.635). Moreover, the meta-analysis showed no significant difference in the incidence of maternal complications between the patients exposed to IFN-α and those not exposed (OR 0.60, 95% CI: 0.09–3.83; *P* = 0.588), with a pooled estimate of 3.8% (95% CI: 0.00–0.168) (Figure 2).

### Meta-Analysis of Pregnancy Outcomes After IFN Exposure

As a member of type I IFNs, IFN-β also presents the function of antivirus, immune regulation, and antiproliferative activity, which is attributed to the similar structure and same receptors with IFN-α. Hence, we further evaluated the influence of IFN exposure on outcomes in pregnant women exposed to any type of IFNs.

**Figure 3:**
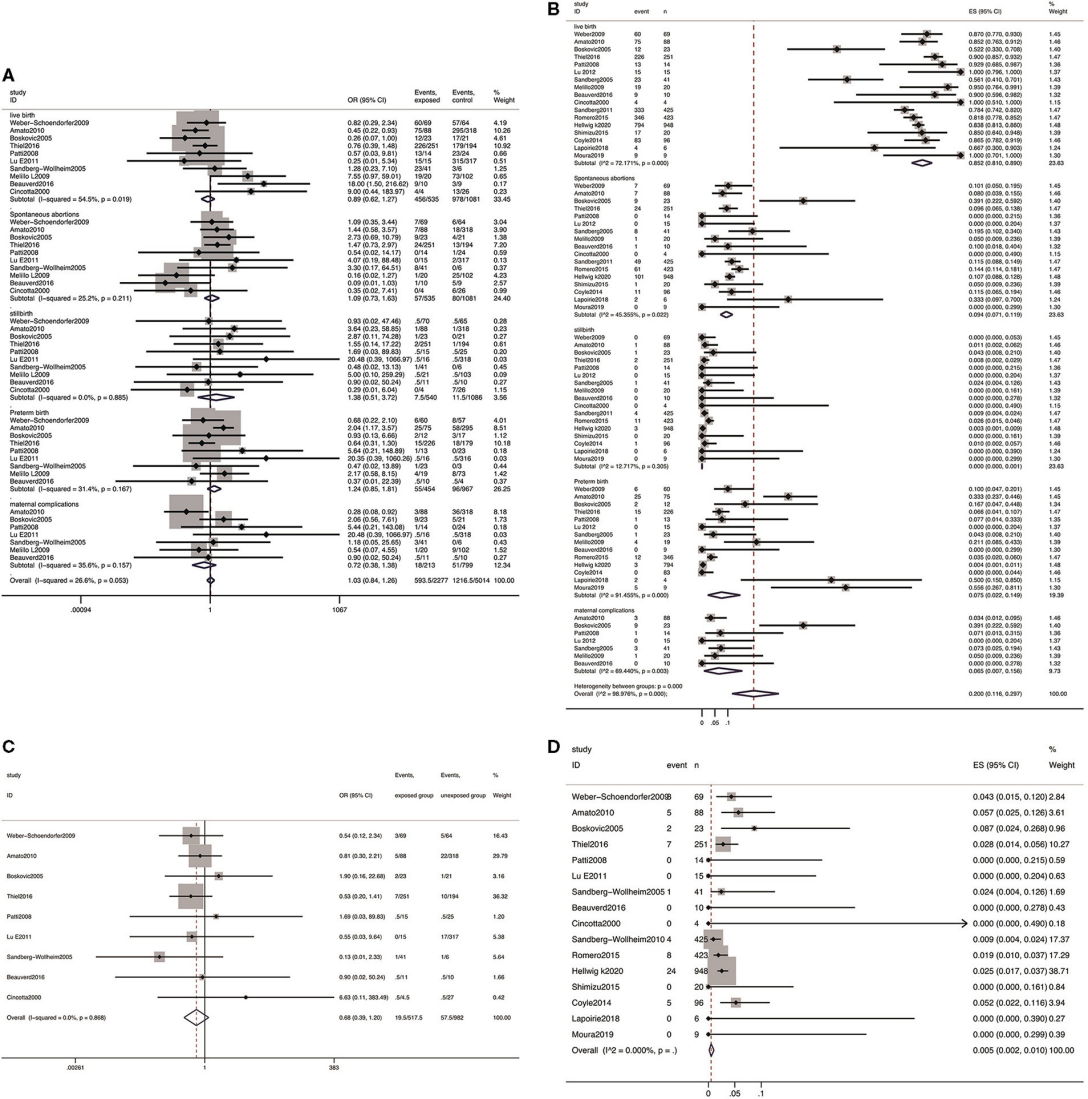
Meta-analysis of pregnancy outcomes after IFN exposure. **(A)** The OR of pregnancy outcomes in IFN-exposed group compared with un-exposed group; **(B)** the pooled rate of each pregnancy outcome in IFN-exposed patients; **(C)** the OR of birth defects in IFN-exposed group compared with un-exposed group; and **(D)** the pooled rate of birth defects in IFN-exposed patients.

All 17 studies reported the incidence of live birth with a total of 2,462 pregnancies with IFN exposure, the pooled rate was 85.2% (95% CI: 0.81–0.89). Compared with patients unexposed to IFNs, it showed that IFN did not present adverse effect on live birth (OR 0.89, 95% CI: 0.62–1.27, *P* = 0.514) (Figure 3A).

The pooled estimates showed no increased risk of spontaneous abortion (OR 1.09; 95% CI: 0.73–1.63; *P* = 0.672), stillbirth (OR 1.38, 95% CI: 0.51–3.72; *P* = 0.530), preterm delivery (OR 1.24, 95% CI: 0.85–1.81; *P* = 0.260), and maternal complications (OR 0.72, 95% CI: 0.38–1.38; *P* = 0.326) among patients exposed to IFN compared with the patients unexposed to IFN prior to conception or during pregnancy (Figure 3A).

Among a total of 2,462 infants in proportion meta-analysis from 17 studies reporting spontaneous abortion and stillbirth as outcome measures, 282 presented spontaneous abortion, yielding a pooled estimate of 9.4% (95% CI: 0.071–0.119), and 24 presented stillbirth, yielding a pooled estimate of 0.0% (95% CI: 0.000–0.001). From the 14 studies (76/1,688) reporting data on preterm delivery, a pooled estimate of 7.5% (95% CI: 0.022–0.149) was explored. From the seven studies reporting data on any maternal complications, 67 events were observed in 209 pregnancies, and the pooled rate was 6.5% (95% CI: 0.007–0.158).

#### Birth Defect

Nine studies reported the incidence of birth defects (including any birth defects and major birth defects), a total of 18 and 56 cases of birth defects out of 518 and 982 live births in the two groups were reported, respectively. The results showed that there was no obvious distinction in risk of birth defects (OR 0.68, 95% CI: 0.39–1.20; *P* = 0.868; Figure 3C) between patients exposed to IFN and those without any IFN treatment prior to conception or during pregnancy. In addition, the pooled estimate of birth defects was 0.51% (95% CI: 0.003–0.011, *P* = 0.00, Figure 3D) in the group exposed to IFN.

### Publication Bias

Studies included for the final analysis were also graphically assessed for any potential publication bias through a funnel plot (Supplementary Figure). The studies were plotted with the estimated effect on the horizontal axis and the standard error of the estimated effect on the vertical axis. Studies with a smaller sample scatter more widely at the bottom of the graph, while larger studies are closer to the true effect of the intervention and are positioned in the upper part of the diagram. Studies that are more precise fall within the 95% CI. In the absence of a publication bias, the plot should look symmetric.

## Discussion

Since be cloned and large-scale produced, type I IFNs have been successfully applied as therapies in patients suffering from various diseases (44–46). Although there are scant published data discussing the safety in pregnant women with chronic HBV infection, IFN has been recommended widely in MS, ET, chronic myeloid leukemia, multiple myeloma, and others (46–48). Furthermore, with growing research data in pregnant women with MS exposed to IFN-β-1a, the Food and Drug Administration has removed the category C warning from the package insert for IFN-β-1a in 2020, which gives more confidence to women with MS to continuing IFN therapy while starting to grow their families. In this meta-analysis including the most comprehensive and up-to-date research on the safety of INF exposure in pregnant women, we found that exposure to type I IFNs during pregnancy did not adversely affect pregnancy or infant outcomes.

In these meta-analysis enrolled 3,543 pregnancies, the pooled estimate of live birth was 85.2% in IFN-exposed group, which is in accordance with that reported in the general population (49). When further compared with pregnant women with the same diseases unexposed to IFN, there was no significant difference, suggesting no evidence of the adverse effect on live birth. This similarity has also been presented in other systematic literature reviews and observational studies (50, 51). Additionally, a recent meta-analysis about pregnant women with myeloproliferative neoplasms suggests that the use of IFN-β during pregnancy was associated with higher successful pregnancy than observation alone, which may be attributed to the potential antiproliferative properties of IFN in myeloproliferative neoplasms (52). In view of these available research results, IFN exposure displays no adverse impact on successful pregnancy.

In line with the general pregnancy population, spontaneous abortion is the primary adverse outcome in the current investigation. A pooled rate of 9.4% was presented, which is not higher than the estimates based on the U. S. National Survey of Family Growth data (15–16%) (53) and that from the general population (16%) (54). Furthermore, when compared with patients unexposed to IFN, the risk of spontaneous abortion (OR 1.09; 95% CI 0.73–1.63; *P* = 0.672) was not increased. In addition, as presented in Figure 3, the prevalence of stillbirth and preterm delivery remained within the ranges reported in the general population (49). Moreover, the risks of these adverse pregnancy outcomes were not increased after exposure to IFNs in pregnant women with the same diseases. Several clinical trials and reviews have previously evaluated the safety profile of IFN in pregnant women, the majority of the exposure was during the first trimester (22, 51, 55). A bit higher rates of spontaneous abortions, lower birthweight (20, 21), and shorter mean birth length were presented in observational cohorts with a small sample size (20, 21, 55). Whereas, these negative pregnancy outcomes were not demonstrated with IFN exposure in systematic reviews and clinical cohorts with large samples (22, 34, 51). Based on these results, there is no evidence of a signal, suggesting that exposure to IFNs before conception and/or during pregnancy adversely influences spontaneous abortion, stillbirth, preterm delivery, or other adverse pregnancy outcomes.

In the enrolled studies, there were 67 maternal adverse events reporting from 7 cohorts, and pooled analysis did not show an increased risk of maternal complications in pregnant women exposed to IFN. These findings align with the previous meta-analysis concerning the IFN exposure in pregnant women with myeloproliferative neoplasms, and are consistent with the worldwide prevalence (56). Whereas, some IFN-associated adverse events, including general fatigue, hair loss, and IFN-associated autoimmune diseases, as well as other maternal complications including pre-eclampsia, gestational diabetes, were not sufficiently evaluated in the current analysis. It is thought that the main concern of the included studies contributes to whether or not it is reported. Furthermore, the reporting bias may also be due to the missing data associated with retrospective studies.

As the main concern among hepatologists about IFN exposure during pregnancy, the pooled estimate of birth defects was 0.51% in this meta-analysis, which is lower than that reported in the United States (3%) (57) and in the European Union (2%) (58). With the largest prospective cohort providing a safety profile of women with MS exposed to IFN-β, Hellwig and his colleagues reported that the prevalence of birth defects was within an estimated range of 2.1–2.8% (51). The lower estimates of birth defects may be attributed to reporting bias associated with retrospective cohorts, and selective termination due to concern about the “potential effect” of IFNs. Whereas, there have been reports that expression of type I IFNs is constitutive in placental, regulating the immune balance between mother and fetus during gestation (59, 60). IFN-α is reportedly present in fetal blood, amniotic fluid, and placental amniotic membrane to prevent intrauterine viral infection (61). Finally, among the 18 cases with birth defects, no consistent pattern of developmental abnormalities was identified, which suggests that there is no evidence of signal suggesting exposure to type I IFN resulting in birth defects.

As a major choice of antiviral therapy, especially in young patients with HBV, unintended pregnancies during IFN-α treatment is inevitable. Although only case reports were retrieved in pregnant women with viral hepatitis, there were cohorts presenting outcomes from patients with other myeloproliferative diseases. Similar to a previous investigation, comparing with those with same diseases without IFN-α treatment, the risk of adverse outcomes, including spontaneous abortion, stillbirth, preterm delivery, birth defects, and maternal complications, were not increased in pregnancies exposed to IFN-α (52). Although the included sample was small, the results were further confirmed with a meta-analysis concerning pregnancy outcomes after exposure to IFN-α or IFN-β, which shares the same receptor with IFN-α and is also approved for the treatment of viral hepatitis (35). Overall, the current meta-analysis presents a comprehensive assessment of the safety in pregnancies with exposure to type I IFNs and provides references for clinical decisions in unintended pregnancies during IFN treatment.

## Limitations

There are inevitable limitations in the present meta-analysis. First, the included studies in this research were retrospective cohorts and case–control observations, and none were randomized controlled trials. However, from an ethical point of view, it is ethically unacceptable to perform ideal randomized controlled trials in such a special population. In addition, during the process of literature inclusion, we conducted a rigorous quality assessment of the included cohort studies according to NOS, and only those with moderate quality were included in this meta-analysis. Due to the particularity of the study population and intervention conditions, it is difficult to conduct higher-quality studies in the future. Second, there were only case reports about pregnant women with viral hepatitis, and only a few cohorts were eligible for meta-analysis. Because of the conventional concern of the “potential teratogenic effects of IFN” among hepatologists, leading to selective/induced termination, only a few studies are available reporting pregnancy outcomes from women with viral hepatitis. Another limitation of the current meta-analysis is the potential for under-reporting due to retrospective studies. Whereas, prospective observational studies or registries require many years to reach a sample size that afford the statistical power to detect a difference in the relevant risk compared with the general population or untreated patients. Furthermore, due to dose and duration of IFN exposure in every single case was unavailable, we could not determine the association of adverse pregnancy outcomes with higher doses or longer duration of IFN exposure. Nevertheless, this systematic review with comprehensive evaluation of type I IFNs could provide references for hepatologists to make a strategic clinical decision.

## Conclusions

Taken together, the current analysis presented a comprehensive assessment of the safety of IFN exposure during pregnancy. The estimated prevalence and risks of adverse pregnancy outcomes and birth defects had not been worsened with IFN exposure before conception and/or during pregnancy. Despite the low-to-moderate quality of the enrolled studies in this analysis, there is no evidence of signal suggesting termination of pregnancy after exposure to IFN prior to or during pregnancy. However, as with all drugs used in pregnancy, it should be encouraged and supported to collect data on birth outcomes to further assess any potential risk of adverse outcomes.

## Data Availability Statement

The original contributions presented in the study are included in the article/Supplementary Material, further inquiries can be directed to the corresponding authors.

## Author Contributions

MZ, SF, and DR: Study design, study identification, data collection and extraction, quality assessment, data analysis and interpretation, and manuscript drafting. NY, YF, and TN: Data collection, quality assessment, manuscript revision. YC: Study design, data analysis, interpretation, and critical review of the manuscript. TC, YZ, and JL: Study concept, study design, data analysis, interpretation of data, manuscript drafting and revision, quality control of algorithms, and study supervision. All authors contributed to the article and approved the submitted version.

## Conflict of Interest

The authors declare that the research was conducted in the absence of any commercial or financial relationships that could be construed as a potential conflict of interest.

## Publisher's Note

All claims expressed in this article are solely those of the authors and do not necessarily represent those of their affiliated organizations, or those of the publisher, the editors and the reviewers. Any product that may be evaluated in this article, or claim that may be made by its manufacturer, is not guaranteed or endorsed by the publisher.
