# Supplementary material for: Maternal and Fetal Outcomes After Interferon Exposure During Pregnancy: A Systematic Review With Meta-Analysis
Source: Front Reprod Health. 2021 Aug 12;3:702929. doi: 10.3389/frph.2021.702929 (PMC9580814; doi:10.3389/frph.2021.702929)
Supplement: Supplementary file 1 [file Table_1.docx]

**Supplementary table.** Quality assessment of the included cohort studies with the Newcastle-Ottawa Assessment Scale.

| **Study ID** | **Selection** | | | | **Comparability** | | **Outcome** | | | **Total scores** |
| --- | --- | --- | --- | --- | --- | --- | --- | --- | --- | --- |
|  | **1** | **2** | **3** | **4** | **5-1** | **5-2** | **6** | **7** | **8** |  |
| Weber2009^30^ | 1 | 1 | 1 | 1 | 1 | 1 | 1 | 1 | 1 | 9 |
| Amato2010^31^ | 1 | 1 | 1 | 1 | 1 | 0 | 1 | 1 | 1 | 8 |
| Boskovic2005^32^  Thiel2016^33^  Patti2008^34^  Lu2011^35^  Melillo2010^37^  Sanderg2005^36^ | 1  1  1  1  1  1 | 1  1  1  1  1  1 | 1  1  1  1  1  1 | 1  0  1  0  1  0 | 1  1  1  1  0  1 | 0  0  0  0  0  0 | 1  1  1  1  0  1 | 1  1  1  0  1  1 | 0  0  1  0  1  0 | 7  6  8  5  6  6 |

1. Representativeness of the exposed cohort. ②Selection of the nonexposed cohort. ③Ascertainment of exposure. ④Demonstration that the outcome of interest was not present at the start of the study. ⑤Consider the comparability of the exposed group and the unexposed group in the design and statistical analysis: 5-1 study controlled the most important confounding factors; 5-2 study controlled any other confounding factors. ⑥Assessment of outcome. ⑦Follow-up was long enough for outcomes to occur. ⑧Adequacy of follow-up of cohorts
