# Supplementary material for: Maternal and Fetal Outcomes After Interferon Exposure During Pregnancy: A Systematic Review With Meta-Analysis
Source: Front Reprod Health. 2021 Aug 12;3:702929. doi: 10.3389/frph.2021.702929 (PMC9580814; doi:10.3389/frph.2021.702929)

Supplementary Figure. Funnel plot for publication bias of different outcomes. A) Spontaneous abortions, B) Preterm birth, C) Stillbirth, D) Birth defect

Supplementary Figure A


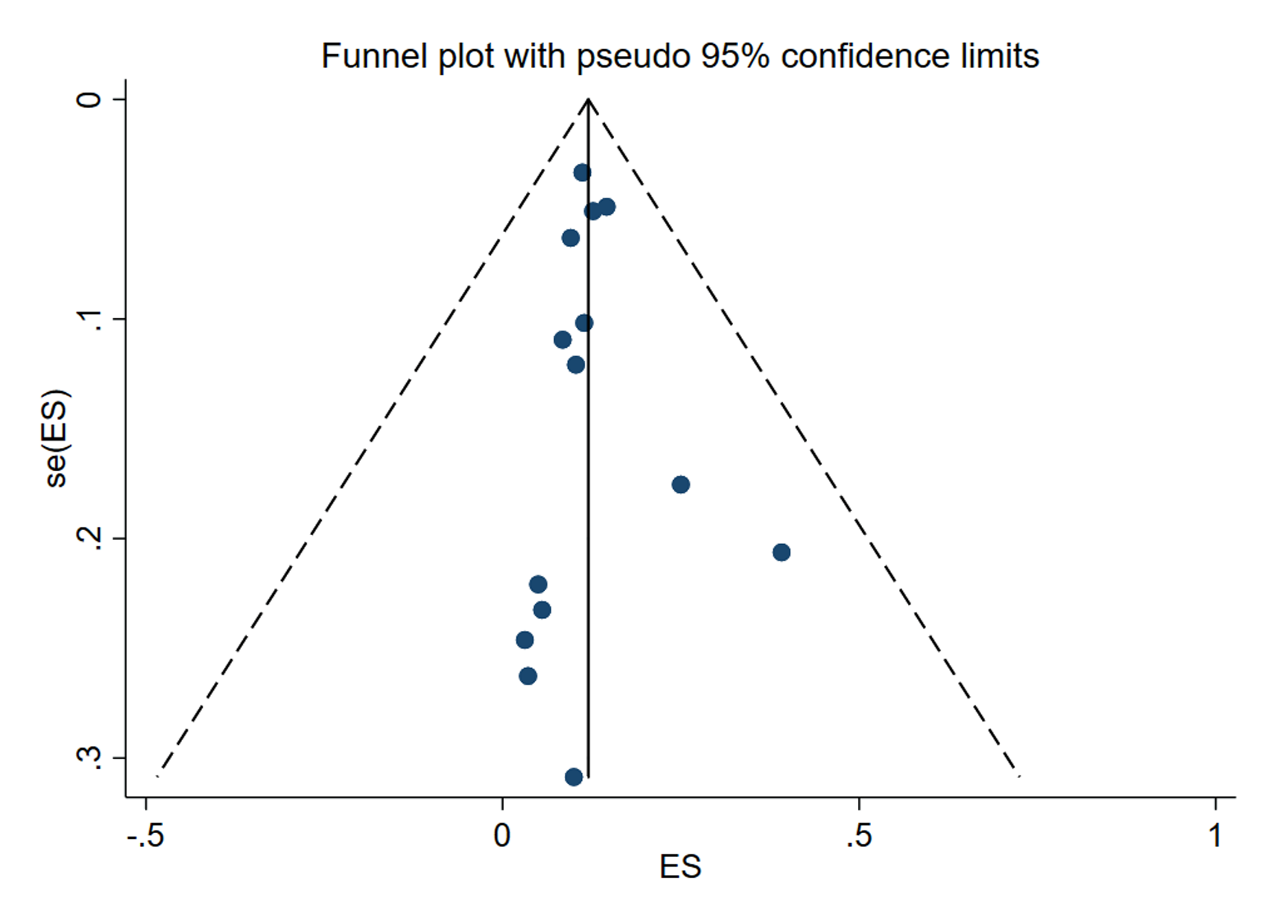


Supplementary Figure B


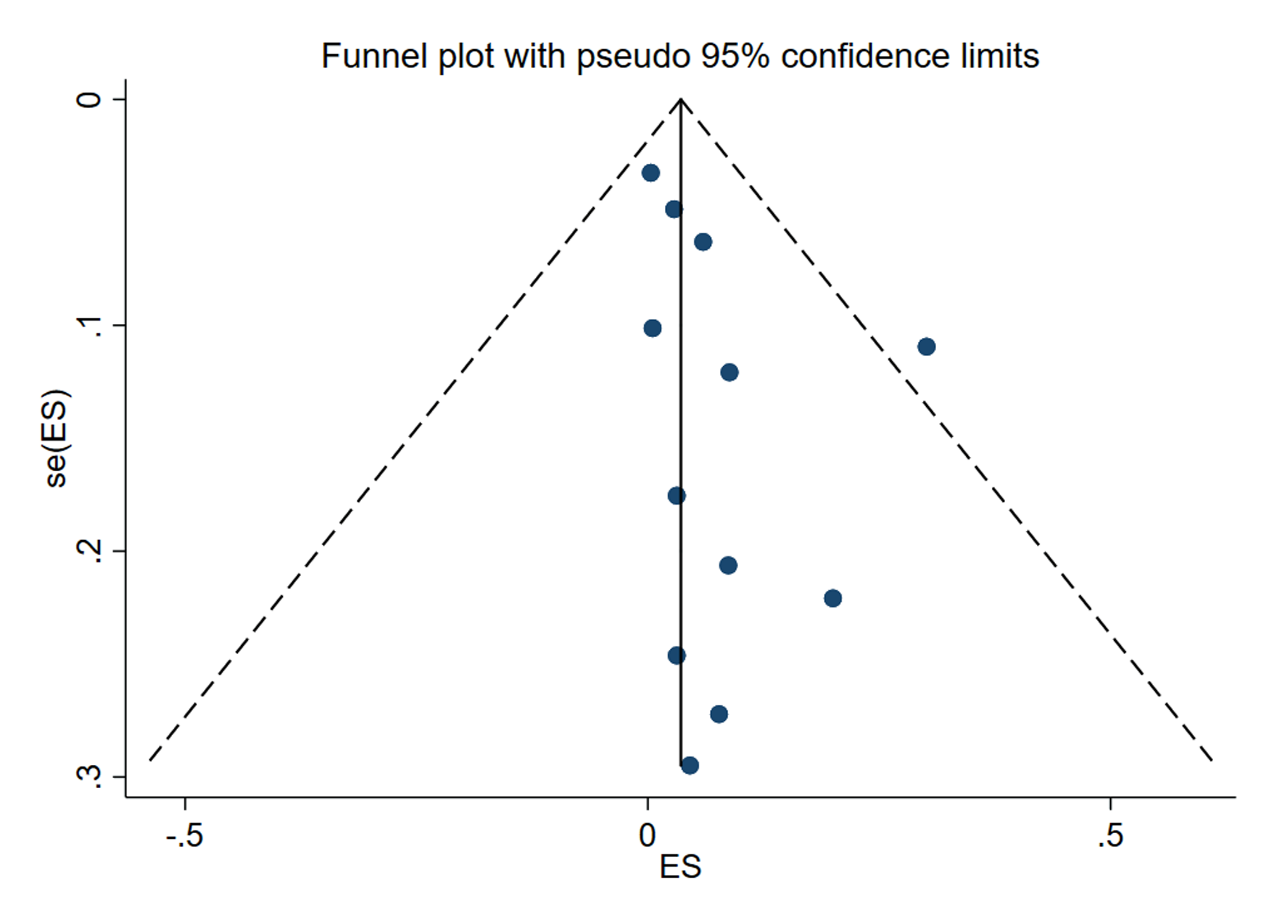


Supplementary Figure C


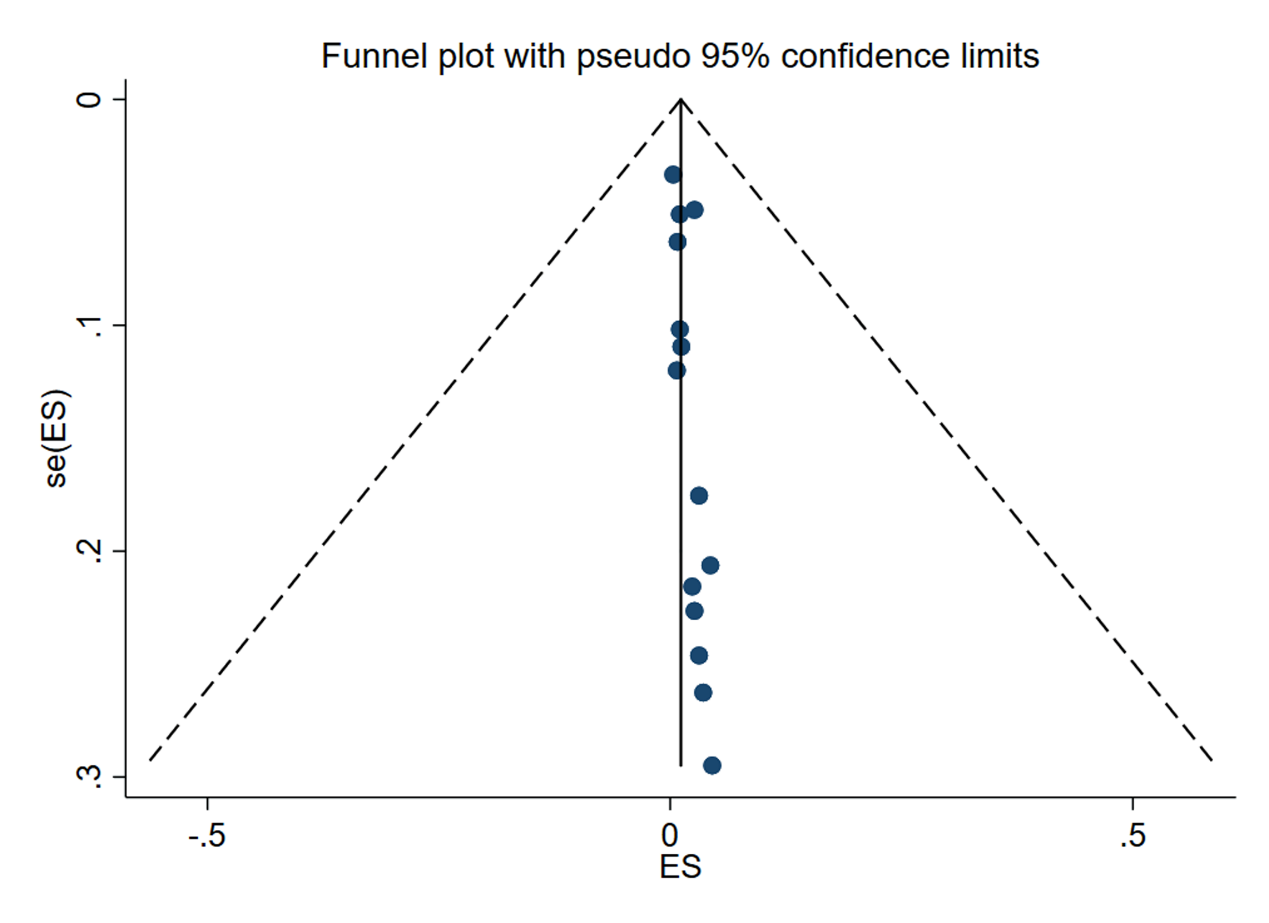


Supplementary Figure D


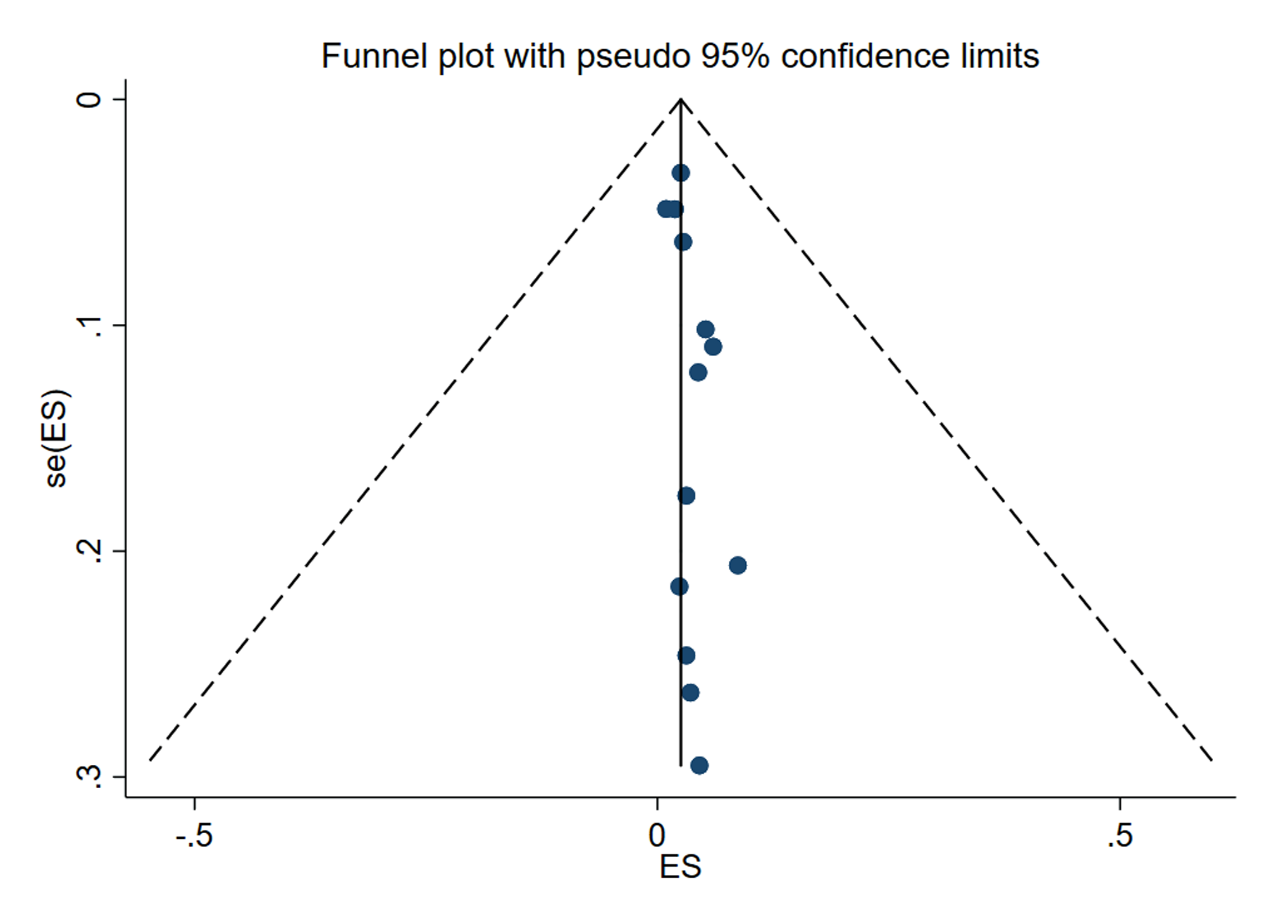

Supplement: Supplementary file 2 [file Data_Sheet_1.docx]
